# Effects and Mechanism of *Ganoderma lucidum* Polysaccharides in the Treatment of Diabetic Nephropathy in Streptozotocin-Induced Diabetic Rats

**DOI:** 10.1155/2022/4314415

**Published:** 2022-03-08

**Authors:** Yu Hu, Shu-Xiang Wang, Fu-Yu Wu, Ke-Jia Wu, Rui-Ping Shi, Li-Hong Qin, Chun-Feng Lu, Shu-Qiu Wang, Fang-Fang Wang, Shaobo Zhou

**Affiliations:** ^1^Basic Medical College, Jiamusi University, Jiamusi, Heilongjiang, China 154002; ^2^School of Medicine, The First Affiliated Hospital of Jiamusi University, Jiamusi 154003, China; ^3^School of Medicine, Huzhou University, Huzhou Central Hospital, Huzhou 313000, China; ^4^School of Life Sciences, Institute of Biomedical and Environmental Science and Technology, University of Bedfordshire, Luton, UK LU1 3JU

## Abstract

*Ganoderma lucidum* polysaccharides (GLP) have renal protection effect but there was no study on the diabetic nephropathy. This study was designed to investigate its effect and mechanism using a diabetic rat model induced by streptozotocin (50 mg/kg, i.p.). The diabetic rats were treated with GLP (300 mg/kg/day) for 10 weeks. The blood glucose, glycated hemoglobin, body weight, and the levels of blood creatinine, urea nitrogen, and urine protein were assessed. And renal pathologies were assessed by the tissue sections stained with hematoxylin-eosin, Masson's trichome, and periodic acid-Schiff. The expression of phosphorylated phosphoinositide 3 kinase (p-PI3K), phosphorylated protein kinase B (p-Akt), and phosphorylated mammalian target of rapamycin (p-mTOR), the autophagy proteins beclin-1, LC3-II, LC3-I, and P62; the apoptosis-related proteins caspase-3 and caspase-9; and the inflammation markers IL-6, IL-1*β*, and TNF-ɑ were assessed. Results showed that GLP alleviated the impairment of renal function by reducing urinary protein excretion and the blood creatinine level and ameliorated diabetic nephropathy. The expression of p-PI3K, p-Akt, and p-mTOR in the diabetic kidney were significantly reduced in the GLP treatment group compared to the without treatment group. GLP treatment activated the autophagy indicators of beclin-1 and the ratio of LC3-II/LC3-I but reduced p62 and also inhibited the expression of caspase-3, caspase-9 and IL-6, IL-1*β*, and TNF-ɑ. In conclusion, the effect of GLP amelioration diabetic nephropathy may be via the PI3k/Akt/mTOR signaling pathway by inhibition of the apoptosis and inflammation and activation of the autophagy process.

## 1. Introduction

Diabetic nephropathy, a most common complication of glomerulosclerosis, is one of the important factors leading to renal failure in diabetic patients [1]. With the increase in newly diagnosed cases and a 5-year survival rate of approximately 20%, diabetic nephropathy has caused widespread concern [2]. Genetic, oxidative stress, hemodynamic abnormalities, and inflammatory responses are all related to the onset of diabetic nephropathy [3–5]. Presently, clinical treatment mainly aims to alleviate kidney injury by controlling blood sugar, blood lipid, and blood pressure; however, this action can only slow down the process of renal failure and cannot prevent or reverse the development of the disease. Therefore, finding an efficacious medication for diabetes and its complications, e.g., diabetic nephropathy, is critical in clinical practice.

*Ganoderma lucidum*, an oriental porous fungus, contains a variety of active substances. *Ganoderma lucidum* polysaccharides (GLP), one of its active ingredients, have unique medicinal and healthcare value and have attracted various research interests [6]. GLP have important biological activities, such as immunoregulation, anti-inflammatory, and antiaging effects, lowering blood glucose, and protecting the liver [6–9], but there is no research on its antinephropathic effect. Zhu et al. [9] isolated and purified a new polysaccharide (PSG-1) from *Ganoderma lucidum* and found that it significantly reduced fasting blood glucose levels, improved endothelium-dependent aorta relaxation, and increased phosphatidylinositol 3-kinase (PI3K), phosphorylated AKT (p-Akt), endothelial nitric oxide synthase (eNOS), and nitric oxide in the aorta of diabetic rats. After binding insulin to its receptor, PSG-1 can also activate the insulin receptor tyrosine kinase and phosphorylate it to produce PI3K [10]. One regulator of mesangial dysfunction in hyperglycaemia is mammalian target of rapamycin (mTOR) which plays a role in the occurrence and development of diabetic nephropathy. Its downstream effectors play a key role in cell growth and hypertrophy, while the inhibition of mTOR by rapamycin can prevent the development of diabetic nephropathy in animals with type 1 and type 2 diabetes [11].

Autophagy is a self-protection mechanism in cells. It can obtain energy by removing and degrading damaged organelles and recycling the biological macromolecules in the cell to maintain the metabolic balance and the stability of the internal environment [12]. Many studies have shown that autophagy is involved in the pathogenesis of diabetic nephropathy, and that it could serve as a new therapeutic target in the treatment of this condition [13]. Autophagy is an intracellular catabolic process, in which lysosomes are involved in the aging and degradation of damaged organelles and proteins, while LC3 and beclin-1 proteins are early marker proteins of autophagy. Apoptosis, also known as programmed cell death, is an important form of cell homeostasis.

Immune system activation and inflammatory response play important roles in the occurrence and development of diabetic nephropathy. Immune cells such as macrophages are involved in prediabetic nephropathy and renal function damage, and monocyte macrophages can also produce cytokines such as IL-6, IL-1*β*, and TNF-*α*. Together with other inflammatory factors, they are involved in the development of diabetic microvascular disease. TNF-*α* is an important proinflammatory cytokine and is part of the acute phase response. It is mainly produced by macrophages and monocytes; however, increased expression of TNF-*α* has been observed in glomeruli and proximal tubular epithelial cells in a diabetic nephropathy model [14]. Through NF-*κ*B signaling, TNF-*α* can induce the transcription of cytokines, thereby, affecting the survival, proliferation, and adhesion of cells and promoting inflammatory responses and apoptosis. The occurrence of apoptosis is regulated by cysteine-specific protease (caspases). Caspase-9 is an important initiating factor in the process of apoptosis, while caspase-3 is the executive factor in the process of apoptosis.

This study intends to further explore the effect of GLP in protecting the kidney of diabetic rats with respect to its effect on the PI3K/Akt/mTOR signaling pathway as well as on autophagy, apoptosis, and inflammation, in order to further evaluate the application of GLP as a new agent in the prevention and treatment of diabetic nephropathy.

## 2. Materials and Methods

### 2.1. Animals and Research Protocols

This study was approved by the Research Ethics Committee of Jiamusi University (No. 216-JMSU). All steps were taken to reduce animal suffering by following the guideline of using laboratory animals by the Chinese Ministry of Science and Technology. Thirty 8-week-old male specific-pathogen-free (SPF) SD rats, weighing 180-220 g, animal license number: SCXK (Lu) 20140007, were purchased from the Animal Experiment Centre of Jiamusi University. All rats were kept in a laminar flow rack of the SPF Laboratory Animal Center of Jiamusi University. They were placed in an environment with sufficient air circulation and a humidity of 40-70% at a temperature of 22-25°C. They were exposed to 12 h of light, fed with ordinary chow, and provided free access to diet and drinking water for a week. Eight rats were used as a blank control, and the other remaining rats were injected with citric acid-sodium citrate buffer solution in the tail vein. After 12 h of fasting, each rat received a single intraperitoneal injection of streptozotocin 50 mg/kg (Sigma company, USA), and they were provided free access to food after injection. Three days later, fasting blood glucose levels were measured using a blood glucose test strip and a Sinocare ultrasimple blood glucose meter (GA-3); 16 rats with blood glucose values greater than 16.7 mmol/L were randomly divided into a diabetic group and diabetic+GLP group, with 8 animals in each group. The animals were provided normal chow.

The diabetic group was administered saline, the diabetic+GLP group was orally administered GLP (300 mg/kg body weight, per day), and the dosage was based on the previous studies used to efficient lower blood glucose [15, 16] as well as the hypolipidaemic effect [17] which also linked to the diabetic nephropathy. Body weight and fasting blood glucose levels were monitored weekly. After 10 weeks, the rats were placed in a metabolic cage individually to collect urine for 24 h. Subsequently, the rats were euthanized with isofluorane (3% for induction, 2% for maintenance), blood was collected from the eyeballs and centrifuged at 3000 × g for 15 min at 20°C, the serum was separated from the cells, and the samples were stored at -80°C for further analysis. The left kidney was removed and placed in fixation solution for histological and immunohistochemical staining, and the right kidney was stored at -80°C for analysis including western blot detection.

### 2.2. Urine Protein, Serum Creatinine, and Blood Urea Nitrogen Measurement

Renal function was evaluated by measuring renal function indicators, including 24 h urine protein, serum creatinine, and blood urea nitrogen. All kits (Cat. C035-2, C011-2 and C013-2, respectively) were bought from Nanjing Jiancheng Bioengineering Institute (Nanjing, China), and the measurements were performed according to their instructions. The urine protein measurement is based on Microprotein Coomassie Brilliant Blue method. A urinary protein is introduced as a calibrator to improve the determination. Serum creatinine was measured based on colorimetric assay. Creatinine can be catalyzed by sarcosamine hydrolases and generates creatine which can be hydrolyzed into sarcosine and urea by creatine amine hydrolase. The sarcosine is broken down by sarcosine oxidase to generate glycine, formaldehyde, and hydrogen peroxide. The reaction between hydrogen peroxide, 2,4-(6-tri-iodine-3- hydroxybenzoic acid) and 4-ampyrone can be catalyzed by peroxidase and form pink compound. Its optical density value at 515 nm can be used to calculate creatinine content. Blood urea nitrogen was measured by diacetyloxime colorimetry, and urea can react with diacetyl to form red diazine compound. The depth of color is proportional to the content of urea.

### 2.3. Hematoxylin-Eosin (H&E) Staining

Partial tissue of rat kidney was collected, fixed with 4% formaldehyde solution, and gradually dehydrated with a gradient of 75%, 80%, 95%, and 100% ethanol. Next, the tissue was embedded in paraffin, and sections with 3 *μ*m thickness were cut using a microtome. After the xylene dewaxing treatment, the sections were washed with absolute ethanol, followed by washing with distilled water for 2 min. Next, the sections were stained with hematoxylin for 5 min, washed with tap water for 1 min, subjected to hydrochloric acid-ethanol differentiation for 15 sec, soaked in warm water at 50°C for 3 min, stained with eosin for 1 min, and dehydrated with gradient ethanol and xylene medium. They were sealed with dry gum when they were dried and, subsequently, observed under a ×400 microscope (M165FC; Leica) to observe the pathological structure of rat kidneys. The cytoplasm was stained pink, and the nuclei were stained blue.

### 2.4. Masson Staining

Similar to the procedure described in the section of H&E staining, the paraffin section specimens were dewaxed using the conventional method with xylene, stained in hematoxylin solution for 5-10 min, washed with running water; subjected to 1% hydrochloric acid differentiation, rinsed with running water for a few minutes, immersed in Masson composite staining solution for 5-10 min, washed with distilled water slightly, treated with 5% molybdophosphoric acid solution for approximately 5 min, followed by direct counterstaining with aniline blue solution for 5 min, treated with 1% glacial acetic acid for 1 min, dehydrated with 95% alcohol and absolute ethanol separately, rendered transparent using xylene, and sealed with neutral gum. The sections were observed under an optical microscope; the procedure for image acquisition was the same as that in the section of H&E staining.

### 2.5. Periodic Acid-Schiff (PAS) Staining

The kidney tissues were fixed in Carnoy fixative solution at room temperature for 48 h. The paraffin-embedded tissues were cut into 4 *μ*m sections, dewaxed, and washed with distilled water, oxidized with 1% periodic acid for 7 min, washed with distilled water, and immersed in Schiff's solution for 7 min in a dark environment at room temperature. Next, they were rinsed with running water for 10 min. The nucleus was lightly stained with alum hematoxylin staining solution for 2-3 minutes, slightly differentiated with 0.5% hydrochloric acid-alcohol solution after overstaining, rinsed with running water for 10 min, dehydrated with gradient ethanol, rendered transparent using xylene, and sealed with neutral gum. The sections were observed under an optical microscope; the procedure for image acquisition was same as that in Section of H&E staining.

### 2.6. Western Blotting (WB) and Immunohistochemical Staining (IHC)

Western blotting was used to detect the expressions of proteins involved in the PI3K/Akt/mTOR signaling pathway and those associated with autophagy, inflammation, and apoptosis. Frozen kidney tissue was weighed and placed in a glass grinder. Next, 400 *μ*L of RIPA was added for every 50 mg of kidney tissue to a lysis solution (containing 4 *μ*L of phosphatase inhibitor and 4 *μ*L of protease inhibitor); the supernatant was collected after centrifugation at 12000 × g for 30 min. Total protein was extracted using the BCA protein quantitative method according to the instructions provided for the protein extraction kit (Boster, Wuhan, China). The concentration of each protein sample was adjusted to the same, and then 10 *μ*L of the sample was loaded into the gel for electrophoresis. The protein samples were separated by SDS-PAGE, and the proteins in the gel were electrically transferred to a polyvinylidene fluoride (PVDF) membrane. In 5% bovine serum, the nonspecific blots in the membrane were blocked. The PVDF membrane was incubated with the antibody at 4°C overnight and then treated with the corresponding horseradish peroxidase secondary antibody (goat anti-rabbit AB20718) (Goat anti-mouse AB67 898). The membrane was incubated at room temperature for 30 min and then placed in an incubator for 1 h. Next, images were recorded and analyzed after the membrane was washed (Tanon 5200). The main antibodies used were as follows: p-AKT (#4060, working solutions, for IHC, 1 : 1000), AKT (#4691, IHC, 1 : 300), p-mTOR (#5536, IHC, 1 : 50), mTOR (#2983, IHC, 1 : 100), and LC3 (#83506, WB, 1 : 1000; IHC, 1 : 800) from Cell Signaling Technology (Danvers, MA, USA) and p-PI3K (ab182651, IHC, 1 : 200), PI3K (ab191606, IHC, 1 : 500), beclin-1 (ab207612, IHC1:200), p62 (ab56416, IHC, 1 : 800), caspase-3 (ab13847, IHC1 : 500), caspase-9 (ab52298, IHC, 1 : 50), IL-6 (ab9324, IHC, 1 : 250), IL-1*β* (ab9722, IHC, 1 : 100), TNF-ɑ (ab6671, IHC, 1 : 100), and brain natriuretic peptide (BNP) (ab19645, IHC, 1 : 1000) from Abcam (Cambridge, UK). For western blot results of LC3, two bands of LC3I and LC3II generated according to protocol from Cell Signaling Technology.

Kidney tissue specimens were fixed with 4% formaldehyde, rinsed with tap water for 5 min, dehydrated with gradient ethanol, rendered transparent with xylene, and dipped and embedded in paraffin. The paraffin specimens were cut into 2 *μ*m sections. After the paraffin sections were dewaxed and washed with water, they were incubated with 3% H_2_O_2_ and incubated at room temperature for 20 min to eliminate endogenous peroxidase activity. After washing them 3 times with distilled water, 50 *μ*L of primary antibody working solution was added to cover the tissue section. Next, the box was covered with a lid to prevent evaporation and incubated at room temperature for 60 min. Subsequently, the sections were rinsed 3 times with PBS, treated with two drops of secondary antibody solution, incubated at room temperature for 30 min, and rinsed thrice with PBS. They were stained using *Dolichos biflorus* agglutinin, incubated for 5 min at room temperature, and rinsed 3 times with PBS for 5 min each; 2 drops of hematoxylin solution were added into the tissue section. The sections were rinsed with running water after 5 min and then treated with 0.5% hydrochloric acid ethanol after approximately 10 sec, rinsed with tap water, dehydrated with gradient ethanol, rendered transparent with xylene, sealed with neutral gum, and observed under a light microscope. Six sliced section specimens were taken from each group; they were judged as positive by the appearance of clear light yellow/tan particles, and the dye signal in each selected glomerulus or tubulointerstitial region of the images was highlighted using Image Pro Plus quantitative software and quantified.

### 2.7. Data Analysis

Raw data was deposited in the reference [18]. Statistical analysis results were collected from three replicated independent experiments. Data are expressed as the mean ± standard deviation (SD). For statistical comparison, a one-way analysis of variance (ANOVA) was performed on the parameter data. Statistical analysis was performed using GraphPad Prism 6 software (GraphPad, San Diego, California, USA). *p* value was calculated by one-way analysis of variance or multiple *t*-test. *p* < 0.05 was statistically significant.

## 3. Results

### 3.1. Effect of GLP Treatment on Blood Glucose and the Function of Kidney

Changes in the fasting blood glucose during the seventy-day experimental period were monitored weekly to assess the progress of diabetes. At end of experiment, serum glycated hemoglobin, 24 h urine protein, urea nitrogen, and serum creatinine were measured in order to assess the renal function. Rats with fasting blood glucose levels raised above 16.7 mmol/L after streptozotocin injection were used for the treatment with and without GLP. Compared to the diabetic group that was not administered treatment, the group administered GLP treatment showed significantly reduced fasting blood glucose at the ninth week till the end of experiment, even though it did not reach the normal level with GLP treatment alone (Table 1). At the end of the experiment, GLP treatment significantly decreased the level of glycated hemoglobin, 24 h urine protein, blood urea nitrogen, and serum creatinine, which were all significantly increased in the diabetic rats (Table 1). These changes indicate that GLP treatment could significantly improve the kidney function of the diabetic rats even though GLP could not reverse the increased biomarker levels back to normal.

### 3.2. Effect of GLP Treatment on the Histology of Diabetic Nephropathy

**Figure 1:**
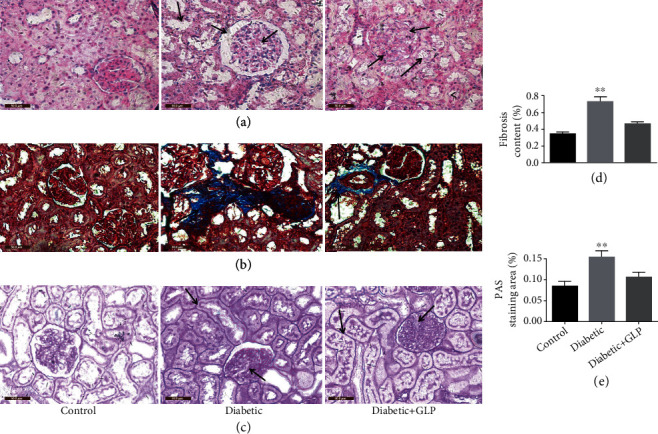
Pathological changes in the kidney tissue of diabetic rats. Representative images showing kidney tissue sections after (a) hematoxylin and eosin, (b) Masson's trichrome, and (c) periodic acid–Schiff staining (PAS). Quantitative results for (d) collagen accumulation assessed using Masson's trichrome staining and (e) extracellular matrix accumulation assessed using PAS staining for the different groups (original magnification ×400). The arrow in the kidney section in the diabetic group ((a), middle) indicates the irregularly arranged cell membranes, disturbed structures, disappeared nucleus, or unclear intercellular boundary. The arrow in the middle image of (b) indicates considerable deposition of collagen fibers, and the arrow in middle image of (c) indicates considerable deposition of reactive glycogen. Values are presented as the mean ± SE; *n* = 4 per group. ^∗∗^*p* < 0.01 versus either the control group or the diabetic+GLP group using Tukey's test. Control: 5 ml/kg saline (p.o.); diabetic: 50 mg/kg streptozotocin (intraperitoneal) and 5 ml/kg saline (p.o.); diabetic+GLP: 50 mg/kg streptozotocin (intraperitoneal) and 300 mg/kg *Ganoderma lucidum* polysaccharides (GLP) (p.o.).

To further analyze the mechanism of GLP in the improvement of the renal function, pathological changes in the kidney specimens were examined by staining with H&E, Masson, or PAS stains (Figures 1(a)–1(c)). The kidney extracellular matrix content via Masson staining (Figure 1(d)) and glycogen or mucin accumulation via PAS staining (Figure 1(e)) were analyzed. Compared to the control group, both were increased significantly in the diabetic group but were reduced significantly after GLP treatment.

The kidneys of the diabetic group that was not administered treatment showed hypertrophy through naked eye observation and felt harder in texture compared to those treated with GLP and the normal groups. In the micrograph of H&E-stained specimens, the glomeruli and kidney tubules of the control group were seen clearly with normal histological structures. However, in the diabetic group, thickened glomerular basement membranes were observed; furthermore, the glomerular cavities were enlarged with irregular outline, the cells in the bulb were unevenly distributed, more degenerated fat was presented, some kidney tubules showed atrophy and collapse, and the cell walls were arranged irregularly (arrows indicate typical changes) (Figure 1(a)). Masson staining shows tubulointerstitial fibrosis (collagen fibers, blue) in the diabetic group. However, after GLP intervention, kidney tubular injury was significantly reduced, and mesangial matrix proliferation was significantly reduced (Figures 1(b) and 1(d)). The specimens stained with PAS showed that the glomerular basement membranes of the control group were intact and significantly reduced extracellular matrix depositions that were observed (Figures 1(c) and 1(e)). A large amount of glycogen deposition was observed in the diabetic group. In the tubules and glomeruli, the glomerular mesangial area was enlarged, and the extracellular matrix was significantly increased (the arrows indicate the typical changes). The above pathological changes were significantly improved in the GLP treatment group, and normal appearance was restored in these tissues.

**Figure 2:**
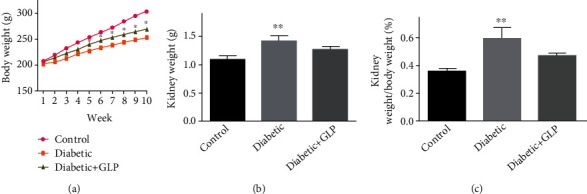
Body weight changes during the experimental period (a) and kidney weight (b) and kidney weight/body weight (%) (c) at the end of the experiment at day 70. Values are presented as the mean ± SE; *n* = 8 per group. ^∗^ compared to the control, both diabetic and diabetic+GLP shows *p* < 0.05 in (a). In (b) and (c), ^∗∗^ indicates *p* < 0.01 versus either the control group or the diabetic +GLP group using Tukey's test. Control: 5 ml/kg saline (p.o.); diabetic: 50 mg/kg streptozotocin (intraperitoneal) and 5 ml/kg saline (p.o.); diabetic+GLP: 50 mg/kg streptozotocin (intraperitoneal) and 300 mg/kg GLP (p.o.).

Body weight in the diabetic group increased slowly, and it was significantly lower compared to the GLP treatment group at week 5; body weight increased continuously and significantly in the GLP treatment group after week 5 (Figure 2(a)), while the body weight increase was the lowest in the diabetic group during the entire experimental period. At end of experiment, both the weight of the kidney (Figure 2(b)) and the ratio of kidney weight to body weight (Figure 2(c)) in the diabetic group were significantly higher than that in the control group; however, this effect was significantly reversed by GLP treatment.

### 3.3. Effect of GLP Treatment on the Level of *Α*-SMA and BNP Expression

**Figure 3:**
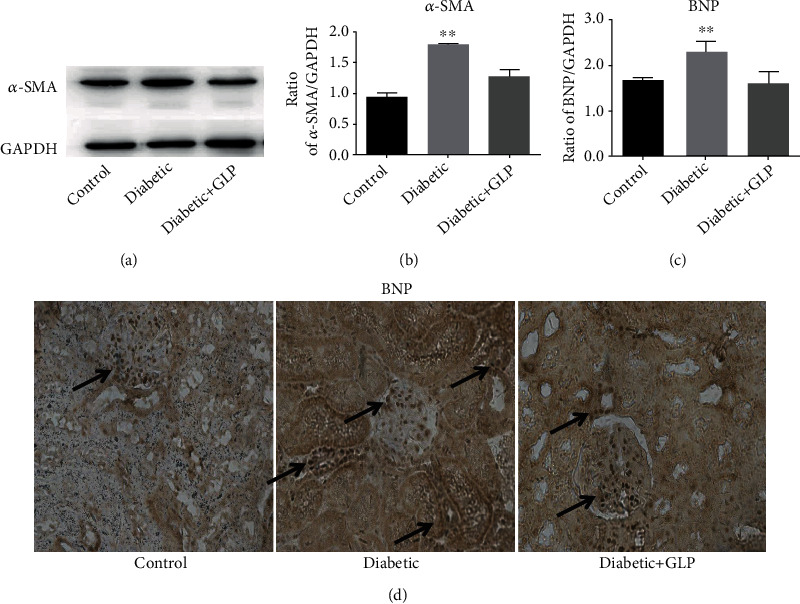
Effect of *Ganoderma lucidum* polysaccharide (GLP) on the protein expressions of *α*-SMA (molecular weight, 42 kDa) assessed by western blot and brain natriuretic peptide (BNP) for immunohistochemical analysis in the kidney tissues of rats from different groups. Micrographs, (d), original magnification ×400; (c) statistic analysis. Representative western blot images (a) and quantitative analysis (b) for expression of *α*-SMA (d) Representative immunohistochemistry micrographs for BNP expression. Strong BNP immunostaining was observed in almost all kidney cells in the diabetic group, compared with the control and diabetic+GLP groups. (c) Statistical analysis of immunohistochemistry results for the renal BNP expression. The arrows in the kidney section in the diabetic group indicate BNP highly expressed areas. In control and treatment groups, BNPs are mainly expressed in the glomerular areas, but there are higher expression in the tubule area in the diabetic group. Values are expression rate represent the mean ± SE; *n* = 3 in each group. ^∗∗^*p* < 0.01 versus the control group and the diabetic+GLP group using Tukey's test. Control: 5 ml/kg saline (p.o.); diabetic: 50 mg/kg streptozotocin (i.p.) and 5 ml/kg saline (p.o.); diabetic+GLP: 50 mg/kg streptozotocin (i.p.) and 300 mg/kg GLP (p.o.).

The level of *α*-SMA expression in the kidney tissue was assessed by western blotting (Figures 3(a) and 3(b)), while the level of BNP expression in the kidney tissues was assessed by immunohistochemical detection (Figures 3(d) and 3(c)). The results showed that the expression of both *α*-SMA and BNP in the kidney tissues of diabetic rats increased significantly compared with that in the control group (*p* < 0.01); however, after GLP intervention, the expression level of both *α*-SMA and BNP in the kidney tissues of diabetic rats decreased significantly, and the difference in these values between the diabetic+GLP group and the diabetic group was statistically significant (*p* < 0.01).

### 3.4. Effect of GLP Treatment on the PI3K/Akt/mTOR Signaling Pathway

**Figure 4:**
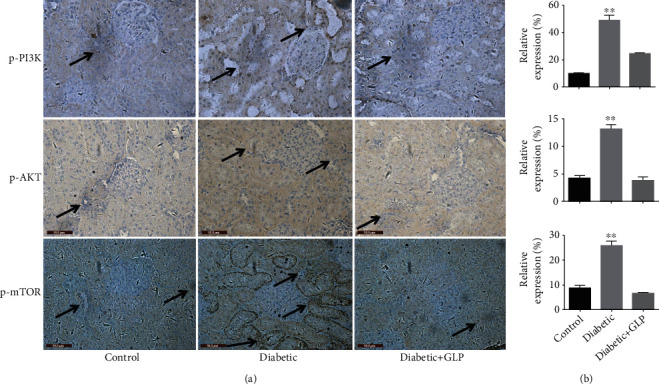
Effect of *Ganoderma lucidum* polysaccharide (GLP) on the protein expression of p-PI3K, p-Akt, and p-mTOR according to immunohistochemical results (a) and their statistical analysis (b) in renal tissues in different groups. (a) micrographs, magnification ×400. Arrows indicate the changed areas. Values represent the mean ± SE; *n* = 3 in each group. ^∗∗^*p* < 0.01 versus the control group and the diabetic+GLP group using Tukey's test. Control: 5 ml/kg saline (p.o.); diabetic: 50 mg/kg streptozotocin (i.p.) and 5 ml/kg saline (p.o.); diabetic+GLP: 50 mg/kg streptozotocin (i.p.) and 300 mg/kg GLP (p.o.).

The level of p-PI3K, p-Akt, and p-mTOR expression in the kidney tissue was assessed by immunohistochemical detection (Figures 4(a) and 4(b)), respectively. The expression of p-PI3K, p-Akt, and p-mTOR in the kidney tissue of the diabetic group was significantly increased compared with that of the normal group (*p* < 0.01). The expressions of these proteins in the kidney tissues of diabetic rats were significantly reduced after GLP intervention (*p* < 0.01).

### 3.5. Effect of GLP Treatment on the Expression LC3-II/LC3-I, Beclin-1, and P62

**Figure 5:**
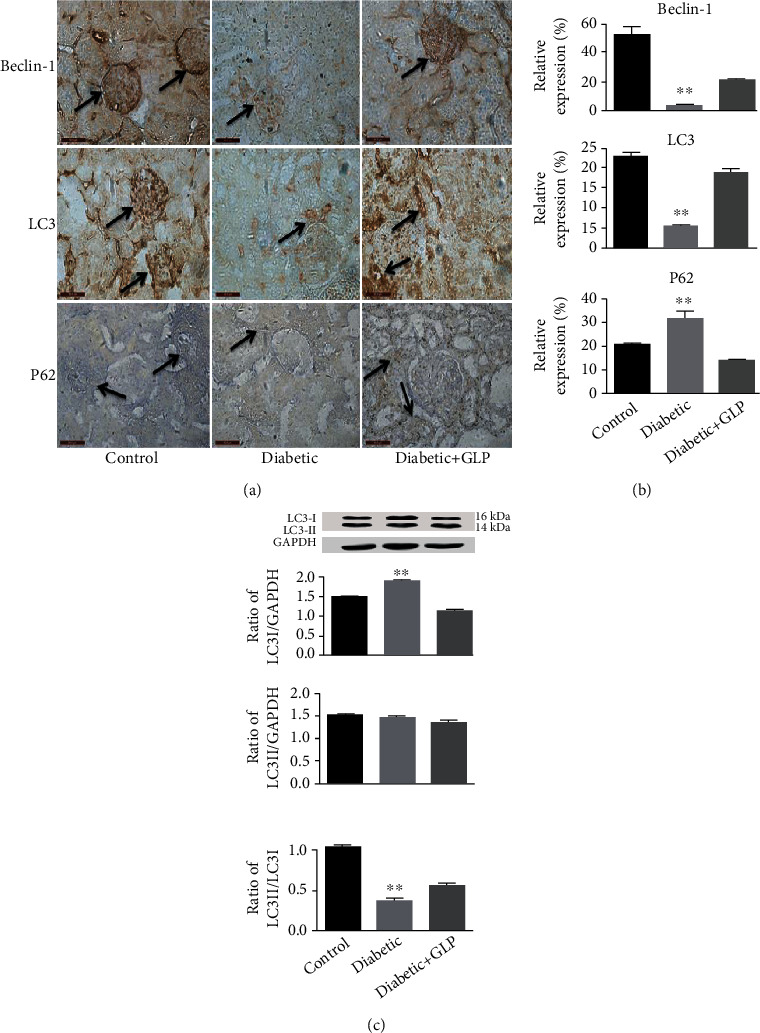
Effect of *Ganoderma lucidum* polysaccharide (GLP) on the expression of beclin-1, LC3, and P62 ((a) immunohistochemical micrographs and (b) their statistical analysis) as well as the LC3-I and II (western blot results, (c)). (a) micrographs, magnification ×400. Arrows indicate the changed areas. Values in (b) and (c) represent the mean ± SE; *n* = 3 in each group. ^∗∗^*p* < 0.01 versus the control group and the diabetic+GLP group using Tukey's test. Control: 5 ml/kg saline (p.o.); diabetic: 50 mg/kg streptozotocin (i.p.) and 5 ml/kg saline (p.o.); diabetic+GLP: 50 mg/kg streptozotocin (i.p.) and 300 mg/kg GLP (p.o.).

The immunohistochemical results of beclin-1, LC3, and P62 were shown in Figure 5(a), and the LC3 was further analyzed by western blot to see the subtype of LC3-II and LC3-I expression, which were shown in Figure 5(c). Compared with the control group, the expression of both beclin-1 and LC3 as well as the ratio of LC3-II/LC3-I in the kidney tissue of the diabetic group was significantly decreased, and the expression of P62 was significantly increased (*p* < 0.01). However, after GLP intervention, beclin-1 and LC3 as well as the ratio of LC3-II/LC3-I were all significantly increased, and the expression of P62 was significantly reduced (*p* < 0.01).

### 3.6. Effect of GLP Treatment on the Expression of Caspase-3 and Caspase-9

**Figure 6:**
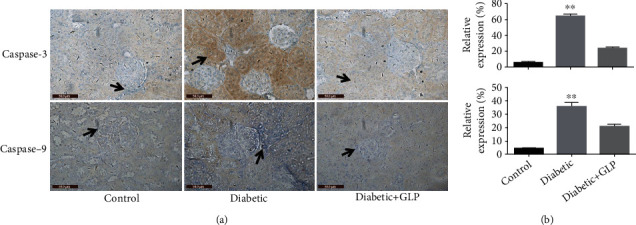
Effect of *Ganoderma lucidum* polysaccharide (GLP) on the protein expression of caspase-3 and -9 in renal tissues according to immunohistochemical micrographs ((a) magnification ×400, arrows indicate the changed areas) and statistical analysis (b). Values represent the mean ± SE; *n* = 3 in each group. ^∗∗^*p* < 0.01 versus the control group and the diabetic+GLP group using Tukey's test. Control: 5 ml/kg saline (p.o.); diabetic: 50 mg/kg streptozotocin (i.p.) and 5 ml/kg saline (p.o.); diabetic+GLP: 50 mg/kg streptozotocin (i.p.) and 300 mg/kg GLP (p.o.).

The western blot and immunohistochemical results of caspase-3 and caspase-9 were shown in Figures 6(a) and 6(b). Results show that the expression of caspase-3 and caspase-9 in kidney tissues of the diabetic group was significantly increased compared with the control group (*p* < 0.01); however, after GLP intervention, their expressions in the kidney tissues were reduced significantly (*p* < 0.01).

### 3.7. Effect of GLP Treatment on the Expression of TNF-ɑ, IL-6, and IL-1*β*

**Figure 7:**
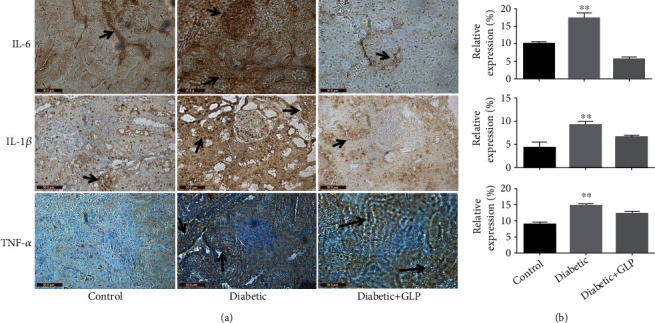
Effect of *Ganoderma lucidum* polysaccharide (GLP) on the protein expression of IL-6, IL-1*β*, and TNF-ɑ in renal tissues according to immunohistochemical micrographs ((a) magnification ×400, arrows indicate the changed areas) and statistical analysis (b). Values are presented as the mean ± SE, *n* = 3 in each group; ^∗∗^*p* < 0.01, compared with either the control group or diabetic+GLP group using Tukey's test. Control: 5 ml/kg saline (p.o.); diabetic: 50 mg/kg streptozotocin (i.p.) and 5 ml/kg saline (p.o.); diabetic+GLP: 50 mg/kg streptozotocin (i.p.) and 300 mg/kg GLP (p.o.).

The immunohistochemical results of the expression of IL-6, IL-1*β*, and TNF-ɑ were shown in Figure 7. In the diabetic group, they were significantly increased compared with the control group (*p* < 0.01); however, after GLP intervention, their expressions were significantly reduced (*p* < 0.01).

## 4. Discussion

Diabetic nephropathy is the most common serious complication of diabetes. It occurs in diabetic patients with a long course, severe illness, and hypertension [19]. Once a large amount of proteinuria occurs, renal function will irreversibly decline progressively and eventually develop into end-stage renal disease. According to statistics, approximately 20-30% of diabetic patients clinically developed diabetic nephropathy [20], and diabetic nephropathy developed into end-stage renal disease, a critical disease, in 20-40% of patients. In China, some traditional Chinese medicines have been recommended to treat diabetic nephropathy [21, 22].

The main pathological features of diabetic nephropathy are glomerular hypertrophy, widened mesangial matrix, and development of fibrosis or sclerosis. The early symptom is the appearance of trace proteinuria, which can lead to kidney damage and failure upon further clinical progression of the disease [23]. Therefore, the production of proteinuria indicates impairment of the structure and function of the glomerular filtration membrane or damaged renal reabsorption. This study found that the 24 h urinary protein, serum creatinine and blood urea nitrogen in diabetic rats were significantly higher than that in the control group. Compared to the diabetic group, they were decreased by 35%, 18%, and 21%, respectively, after GLP treatment, which showed clearly improvement, even though these indices were unable to reach the normal level in the control group (Table 1). These improvements were partly caused by the decrease of fasting blood glucose and glycated hemoglobin by 14% and 16%, respectively. A human study showed an increased 1% of HbA1c which would increase 3 grams (95% CI: 1.5-4.6 grams) heart left ventricle mass which highly affects blood circulation and links to the kidney diseases [24]. In one study [15], GLP (250 mg/kg) could slow down the progression of streptozotocin-induced diabetic nephropathy in mice by decrease blood glucose and triglyceride levels, suggesting the metabolic modulation of GLP. This was also supported by our histological results which kidney tubular epithelial cells were shed, and vacuole degeneration was detected by kidney tissue staining. Inflammatory cells extensively infiltrated the kidney interstitium, kidney tubular hypertrophy and globules were significantly enlarged, the mesangial area was enlarged, and the extracellular matrix was significantly increased. The results were the same as that of previous studies [25]. GLP treatment can maintain a relatively normal structure and the function of kidneys in diabetic rats. In addition, reversal of pathological changes, as observed through H&E, PAS, and Masson staining, suggests that GLP can ameliorate the damaged and pathologically altered kidney tissue. Li et al. [16] showed that GLP (100, 200, and 400 mg/kg) treatment improved streptozotocin-induced diabetic nephropathy and reversed the decreased matrix metalloproteinase-2/tissue inhibitor of metalloproteinase-2 in rats. This further decreased the accumulation of extracellular matrix, suggesting the renoprotective effect of GL-PS through rebalance matrix metalloproteinase-2/tissue inhibitor of metalloproteinase-2.

BNP is a substance composed of 32 amino acids, and it performs multiple functions. It can increase the excretion of sodium from the kidney by acting on the guanylate cyclase receptor, inhibit the sympathetic central nervous system activity and the renin-angiotensin aldosterone system, relax the vascular smooth muscle, and increase endothelial permeability. Recent studies [26] have shown that BNP can inhibit cardiac and vascular remodeling. Another finding [27] showed that BNP increased the risk of renal function in patients with type 2 diabetes. Researches on patients with diabetic nephropathy [28, 29] showed that BNP increased, especially at the early stage of diabetic nephropathy. When fibroblasts transform into myofibroblasts, there is an increase in *α*-SMA expression, migration, and proinflammatory signals and the production of proteins that reshape the extracellular matrix [30]. Meroterpenoids from *Ganoderma* cochlear, cochlearols A and B and polycyclic meroterpenoids, exerted renoprotectivity by inhibiting the expression of renal fibrosis related markers such as collagen I, fibronectin, and *α*-SMA in a dose-dependent manner (5, 10, and 20 *μ*M) [31]. The decreased expression of BNP and *α*-SMA after GLP treatment indicates that GLP plays an important role in kidney protection. This observation was confirmed through the assessment of H&E staining, Masson staining, and PAS staining, which showed that pathological changes in the kidney and kidney fibrosis in diabetic rats were significantly reduced after the GLP treatment.

In terms of the mechanism, PI3K, Akt, and mTOR are considered to play key roles in the insulin signalling pathway, which regulates glucose uptake and glycogen synthesis [32]. In recent years, the role of the PI3k/Akt/mTOR signaling pathway in the development of diabetic nephropathy has received increasing attention. Lu et al. [33] reported that the activation of the PI3k/Akt/mTOR signaling pathway can accelerate the occurrence and deterioration of kidney fibrosis in diabetic rats. This study found that compared with the control group, the expression of p-PI3K, p-Akt, and p-mTOR increased in the diabetic group had significantly decreased after GLP treatment, indicating that GLP may improve diabetic nephropathy by inhibiting the PI3k/Akt/mTOR signaling pathway. Fibrosis of diabetic nephropathy tissue is consistent with the results from previous studies. PI3k/Akt/mTOR is a very important signaling pathway that regulates cell autophagy. It plays an important role in the signal regulation and molecular mechanism of autophagy. This pathway is also key in other important cellular processes, such as cellular survival, appreciation, growth, and differentiation [34]. The PI3k/Akt/mTOR signaling pathway is currently known as the only inhibitory pathway for autophagy, and its activation can inhibit autophagy. Zhong et al. [35] found that ligustrazine can inhibit the expression of p-PI3K, p-Akt, and p-mTOR in the kidney tissues of diabetic rats, thereby, increasing the expression level of the autophagy marker protein LC3B and the ratio of LC3B-II/LC3B-I. The results of this experiment indicate that the PI3k/Akt/mTOR signaling pathway is activated, and autophagy is inhibited in diabetic nephropathy; however, the signaling pathway is inhibited, and autophagy is reactivated after GLP treatment, indicating that GLP plays a renal protective role. The mechanism may be related to its inhibition of the PI3k/Akt/mTOR signaling pathway, which in turn promotes kidney autophagy.

According to previous research, the apoptosis of kidney cells plays an important role in the occurrence and development of diabetic nephropathy. In recent years, increasing evidence shows that there is an interactive effect between apoptosis and cell autophagy, and that the two are interrelated and mutually regulated. When diabetic nephropathy is stimulated by factors such as high glucose, podocytes can not only discard proteins and organelles through autophagy but also undergo apoptosis. The activities of autophagy and apoptosis may vary during different periods. A literature review to understand the relationship between the two revealed the following two aspects: autophagy involves autophagy genes, e.g., ce, which is upstream of the apoptosis process; these genes initiate apoptosis, and their expression affects the degree of apoptosis [36]. Autophagy can maintain the stability of the cell environment and inhibit apoptosis through multiple pathways [37]. Studies have found that normal autophagy plays an important role in reducing the cerebral ischemia-reperfusion injury via inhibition of apoptosis, while an abnormally high level of autophagy can aggravate this injury [38]. Another example of the protective effect of autophagy is demonstrated in intervertebral disc degeneration; activation of autophagy inhibits the degradation of the extracellular matrix of the endplate chondrocytes via inhibition of apoptosis [39]. In terms of apoptosis, autophagy is indispensable, but the typical apoptosis inhibitory proteins, e.g., Bcl-2, can affect this process [40]. The results of this experiment also showed that the expression of the apoptosis-related proteins caspase-3 and caspase-9 increased in the diabetic group and decreased after treatment with GLP, indicating that GLP can activate autophagy, play an antiapoptotic effect, and improve damaged kidney tissue. Autophagy is an innate immune mechanism that can kill pathogens and control inflammation. Autophagy reduces pathogen burden and participates in the processing of antigens to activate adaptive immunity [41]. In recent years, increasing studies have shown that the inflammatory process may play an important role in the pathogenesis of diabetic nephropathy, and adhesion molecules, inflammatory chemokines, and inflammatory factors play specific roles in the development of diabetic nephropathy [42]. Autophagy can negatively regulate inflammatory factors. Lipopolysaccharide (LPS) stimulates the expression levels of IL-1*β* and IL-8 in mice in which the key autophagy gene Atg16L1 was knocked out [43]. In addition, autophagy can reduce the occurrence of inflammation by regulating the composition and activation of inflammatory bodies via regulating the secretion of proinflammatory factors such as IL-1*β* and IL-18 [44]. Kimura et al. [45] showed that renal tubules are one of the most active areas of inflammatory response in chronic kidney injury. Autophagy can effectively reduce damaged and dysfunctional mitochondria by removing damage-related molecules and abnormal lysosomes. Subsequently, this process inhibits the inflammatory response and improves immunoregulation, resulting in protection of renal function. Ma et al. [46] reported that a specific dose of GLP induces a significant effect on diabetic nephropathy, and the mechanism underlying this effect may be through inhibiting the activation of NF-*κ*B/NLRP3 inflammatory bodies, reducing the inflammatory response, and improving renal function, thus restoring the biochemical indicators of diabetes in the blood and urine of mice with nephropathy. Patients with diabetic nephropathy have elevated IL-6, and patients with dominant proteinuria have higher serum albumin levels than patients with microalbuminuria or normal albuminuria [47]. Studies have found [48] that the expression of TNF-*α* is significantly increased in the kidneys of rats with type I diabetes. TNF-*α*, as a key factor that mediates the inflammatory response, can induce an adhesion effect in glomerular endothelial cells. Inflammatory substances cause an increase in the mesangial matrix and mesangial cell proliferation, destroying the glomerular structure [49]. Several extracts of *Ganoderma lucidum* showed amelioration in this progress in a review [50], e.g., one study [51] showed that proteoglycan isolated from *Ganoderma lucidum* fruiting bodies shows protection renal morphology in diabetic mice in a dose-dependently (75, 250, and 450 mg/kg) manner during a 8-week treatment. According to previous research, the mechanism appears also via directly elimination reactive oxygen species, suppresses lipid peroxidation, and indirectly scavenges the radicals via activating antioxidant enzyme systems and chelation with metal ion by forming crossbridge between carboxyl groups decreasing reactive oxygen species generation [52–60]. This study showed that the expressions of the inflammatory factors IL-6, IL-1*β*, and TNF-*α* in the diabetic group were significantly increased, while they were reduced significantly after GLP treatment.

## 5. Conclusion

In summary, these results indicate that GLP produces a significant protective effect in diabetic nephropathy by reducing the renal pathological damage and decreasing blood glucose and glycated hemoglobin and improves the kidney function indicated by decreasing the levels of serum creatinine, blood urea nitrogen, and 24 h urine protein as well as the renal *α*-SMA and BNP expressions and even more GLP increased body weight and kidney weight. GLP inhibited the PI3K/Akt/mTOR signaling pathway and inhibited apoptosis indicators of both caspase-3 and -9 expression. These may be contributed by the activation of autophagy via stimulation beclin-1, LC3-II/LC-I, and reduction the expression of p62. These changes may be used partly to explain the molecular mechanism of GLP on alleviation kidney tissue fibrosis. These effects indicate the potential application of GLP for the treatment of diabetic nephropathy.

## Figures and Tables

**Table 1:** The biochemical indices in different groups at end of experiment, day 70.

Group	Control	Diabetic	Diabetic+GLP	Improvement rate^ (%)
Fasting blood glucose (mmol/L), day 0	4.56 ± 0.34	17.10 ± 1.34^##^	17.77 ± 1.28^##^	
Fasting blood glucose (mmol/L), day 70	4.87 ± 0.27	23.10 ± 1.59^##^	19.98 ± 0.98^##^^∗^	13.51
Glycated hemoglobin (g/L)	1.65 ± 0.10	2.42 ± 0.08^#^	2.03 ± 0.08^##^^∗^	16.12
24 h urine protein (mg/L)	184.6 ± 21.06	372.3 ± 34.57^##^	244.5 ± 32.29^#^^∗^	34.33
Blood urea nitrogen (mmol/L)	4.40 ± 0.48	8.44 ± 0.68^##^	6.70 ± 0.63^#^^∗^	20.62
Serum creatinine (*μ*mol/L)	25.17 ± 1.72	38.00 ± 3.74^#^	31.00 ± 1.41^##^^∗^	18.42

Note: data expressed as the mean ± standard deviation, *n* = 6 in each group. ^Improvement rate was calculated by (diabetic − diabetic − GLP)/diabetic × 100%; ^#^*p* < 0.05, ^##^*p* < 0.01, compared with the control group; ^∗^*p* < 0.05, compared with the diabetic group using Tukey's test. Control: 5 ml/kg saline (p.o.); diabetic: 50 mg/kg streptozotocin (intraperitoneal) and 5 ml/kg saline (p.o.); diabetic+GLP: 50 mg/kg streptozotocin (intraperitoneal) and 300 mg/kg *Ganoderma lucidum* polysaccharides (GLP) (p.o.).

## Data Availability

The data are available here: https://data.mendeley.com/datasets/xf58rsgj94/1 doi:10.17632/xf58rsgj94.1.

## Abbreviations

GLP: *Ganoderma lucidum* polysaccharides
mTOR: Mammalian target of rapamycin
p-Akt: Phosphorylated protein kinase B
PAS: Periodic acid–Schiff staining
PI3K: Phosphoinositide 3 kinase
LC3: Cytoplasmic light chain 3
TNF-ɑ: Tumor necrosis factor–alpha.
